# Prevalence of sexually transmitted infections among pregnant migrant and refugee women: Protocol for a systematic review and meta-analysis

**DOI:** 10.1371/journal.pone.0359424

**Published:** 2026-10-05

**Authors:** Darío S. López-Delgado, Carlos A. Narváez, Gloria L. Chapués-Andrade, Rosa Angela Ruiz Ibarra, Danilo Alejandro Solarte Ordoñez, Andrés Salas-Zambrano, Noah L. Schroeder, Magda Gamba, Lysien I. Zambrano, Oscar H. Franco, Alfonso J. Rodríguez-Morales

**Affiliations:** 1 Hospital Universitario Departamental de Nariño, Pasto, Nariño, Colombia; 2 Universidad Cooperativa de Colombia, Campus Pasto, Colombia; 3 Universidad Nacional Autónoma de Nicaragua, Managua, Nicaragua; 4 University of Florida, Gainesville, Florida, United States of America; 5 Central and University Library of Lucerne, Frohburgstrasse, Lucerne, Switzerland; 6 Faculty of Health Sciences and Medicine, University of Lucerne, Frohburgstrasse, Lucerne, Switzerland; 7 Instituto de Investigaciones One Health, Universidad Tecnológica Centroamericana (UNITEC), Tegucigalpa, Honduras; 8 Health and Bioethics, Julius Center for Health Sciences and Primary Care, Utrecht University, Utrecht, Netherlands; 9 Faculty of Health Sciences, Universidad Científica del Sur, Lima, Peru; 10 Grupo de Investigación Biomedicina, Faculty of Medicine, Fundación Universitaria Autónoma de las Américas-Institución Universitaria Visión de las Américas, Risaralda, Colombia; James Cook University, AUSTRALIA

## Abstract

Sexually transmitted infections during pregnancy are associated with important maternal, fetal, and neonatal complications, including vertical transmission, congenital infection, stillbirth, preterm birth, and increased maternal morbidity. Pregnant migrant and refugee women may face additional risks because displacement, legal precarity, socioeconomic vulnerability, and limited access to antenatal care can delay screening and treatment. However, the burden of sexually transmitted infections in this population remains poorly characterized, and available estimates are fragmented across geographic regions, migration categories, diagnostic methods, and healthcare settings.

**Objective:**

This systematic review and meta-analysis aims to estimate infection-specific prevalence of sexually transmitted infections among pregnant migrant, refugee, asylum-seeking, undocumented, internally displaced, or otherwise displaced women worldwide and to characterize variation by geographic region, migration status, and other clinically relevant factors.

**Methods:**

This protocol is registered in PROSPERO (CRD420251085346). We will include peer-reviewed primary cross-sectional studies and cohort studies with a defined prevalence denominator, while excluding case reports, case series, case-control studies, grey literature and reports without a valid denominator. MEDLINE via Ovid, Embase.com, Scopus, Web of Science, the Virtual Health Library, and the WHO Global Index Medicus will be searched without language, date, or geographic restrictions and citation searches. Two reviewers will independently screen records and extract data using a prespecified form that will be pilot-tested before formal extraction. Risk of bias will be assessed with the Joanna Briggs Institute prevalence checklist. When at least three comparable independent estimates are available, random-effects meta-analysis will be conducted separately for each infection. A participant-level composite of any STI will be analyzed only when explicitly reported using unique individuals and will not be derived by summing infection-specific cases. Certainty will be evaluated using a GRADE-informed approach adapted for prevalence estimates.

## Introduction

Sexually transmitted infections (STIs) remain a significant public health issue, particularly among pregnant women, due to their potential to cause severe maternal and neonatal complications [1,2]. Infections such as syphilis, chlamydia, gonorrhea, and HIV during pregnancy have been associated with miscarriage, premature labor, stillbirth, congenital infections, and increased maternal morbidity and mortality [2,3]. The World Health Organization (WHO) estimates that over one million curable STIs are acquired globally every day, with the highest burden occurring in low- and middle-income countries (LMICs), where access to screening and treatment is limited [4]. Among vulnerable subgroups, pregnant migrants and refugees represent a population at heightened risk due to structural, legal, and socioeconomic barriers that limit their access to antenatal care and STI screening [1,4,5].

Although this increased vulnerability has been recognized, the true burden of STIs in pregnant migrant and refugee women remains poorly characterized. Most studies focus on the general migrant population or non-pregnant women, making it difficult to assess the specific risks during pregnancy. Additionally, inconsistencies in study designs, diagnostic criteria, and population definitions have resulted in fragmented evidence, and many health surveillance systems do not disaggregate data by pregnancy status or migration background [5,6]. The lack of standardized, high-quality data prevents accurate estimation of STI prevalence and limits the development of targeted public health interventions. Moreover, undocumented migrants and asylum seekers are often excluded from research and official data collection, further obscuring the true epidemiological picture [7].

Given the potential consequences of untreated STIs during pregnancy, such as vertical transmission, neonatal sepsis, and fetal loss, this systematic review and meta-analysis aims to estimate infection-specific STI prevalence among pregnant migrant and refugee women and to identify the infections most frequently reported in this population. By consolidating fragmented data while preserving infection-specific denominators, the review seeks to provide regionally informative estimates of disease burden, guide targeted screening strategies, and identify critical evidence gaps in maternal and migrant health.

A conceptual overview of the population of interest, STIs assessed, maternal and fetal/neonatal relevance, methodological workflow, and expected outputs of this systematic review and meta-analysis is presented in Fig 1.

**Fig 1:**
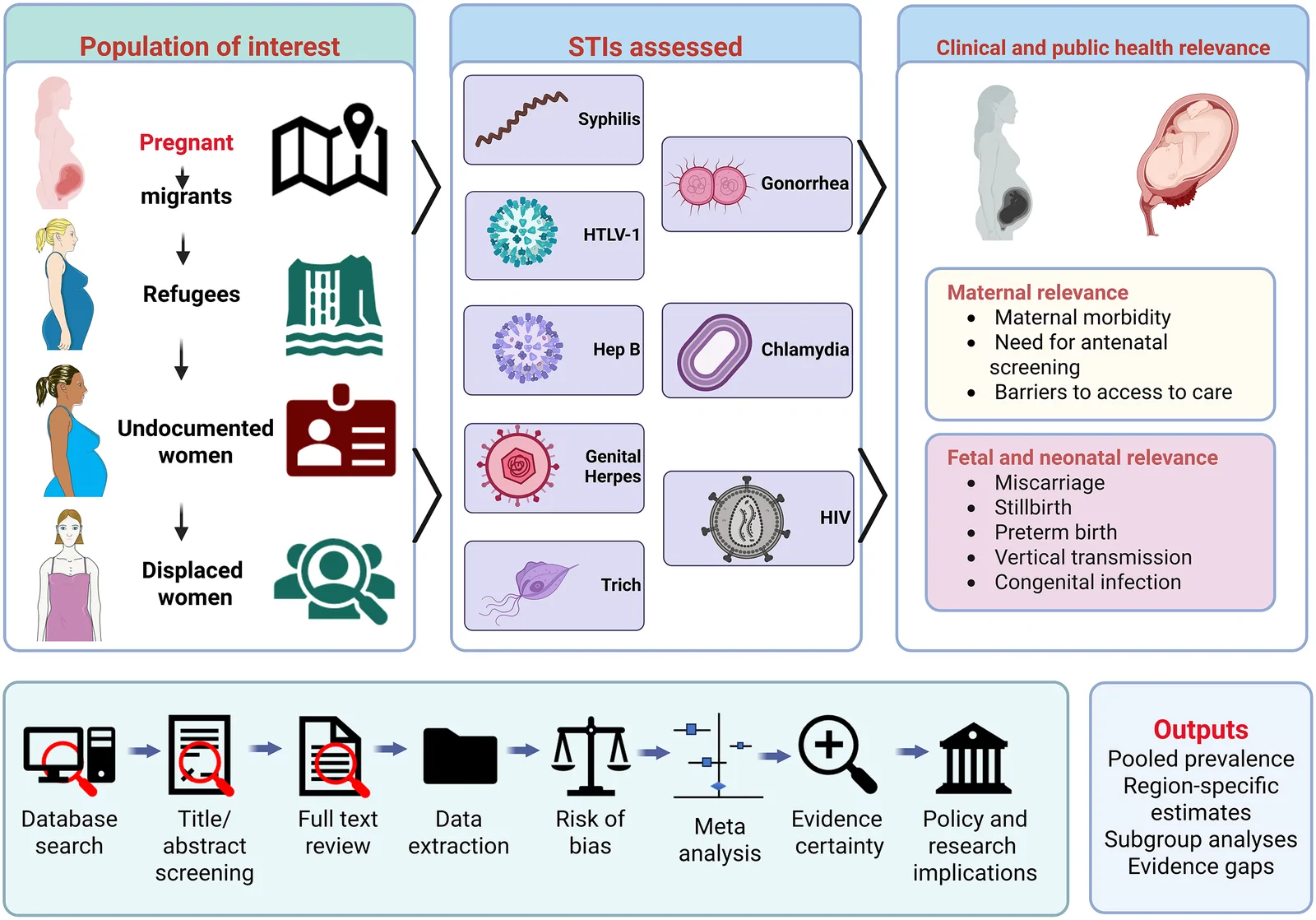
Conceptual overview of the systematic review and meta-analysis. The figure summarizes the population of interest, including pregnant migrants, refugees, undocumented women, and displaced women, the sexually transmitted infections assessed, including syphilis, gonorrhea, HTLV-1, hepatitis B, chlamydia, genital herpes, HIV, and trichomoniasis, and the expected clinical and public health relevance for maternal, fetal, and neonatal outcomes. It also outlines the methodological workflow, from database search, title and abstract screening, full-text review, data extraction, and risk-of-bias assessment to meta-analysis, certainty-of-evidence assessment, and translation of findings into policy and research implications. Expected outputs include pooled prevalence estimates, region-specific estimates, subgroup analyses, and identification of evidence gaps. Abbreviations: HBV, hepatitis B virus; HIV, human immunodeficiency virus; HTLV-1, human T-cell lymphotropic virus type 1; STI, sexually transmitted infection. Created with BioRender.com. Image(s) provided by Servier Medical Art (https://smart.servier.com), licensed under CC BY 4.0 (https://creativecommons.org/licenses/by/4.0/).

## Objectives

### Main objective

1. To systematically review and synthesize the available evidence on infection-specific STI prevalence among pregnant migrant and refugee women.

### Specific Objectives

1. To identify the most frequently reported STIs among pregnant migrant and refugee women.
2. To estimate pooled infection-specific prevalence for each STI when quantitative synthesis is appropriate.
3. To estimate prevalence across geographic regions and migration categories and explore other prespecified sources of heterogeneity when data permit.
4. To assess risk of bias and certainty of the infection-specific evidence and identify methodological and evidence gaps relevant to maternal and migrant health.

## Methods

This systematic review and meta-analysis protocol was registered in the International Prospective Register of Systematic Reviews (PROSPERO) under registration number CRD420251085346. The protocol follows the Preferred Reporting Items for Systematic Review and Meta-Analysis Protocols (PRISMA-P) statement [8]. The completed PRISMA-P checklist is provided in the Supporting Information. The review will follow methodological guidance for systematic reviews and meta-analyses [9], prevalence-focused evidence synthesis [10], and PRISMA 2020 reporting recommendations [11].

The review question, eligibility criteria, search strategy, screening process, data extraction, risk-of-bias assessment, certainty assessment, and quantitative synthesis were prespecified before formal data extraction. Amendments introduced in response to peer review will be dated, documented in the final review, and reflected in the PROSPERO record before resubmission.

### Inclusion and exclusion criteria

This systematic review will include peer-reviewed primary cross-sectional studies and cohort studies that permit estimation of prevalence at a defined assessment. Case reports, case series, case-control studies, qualitative studies, reviews, grey literature and reports without a valid prevalence denominator will be excluded. Predefined eligibility criteria are summarized using the PECOS framework in Table 1. The operational definitions for the terms used in this study are presented in Table 2.

**Table 1:** Eligibility criteria for study selection.

Domain	Inclusion criteria	Exclusion criteria
Population	Pregnant women identified as migrants, refugees, asylum seekers, undocumented migrants, internally displaced persons (IDPs), or otherwise displaced women, regardless of country of origin or host country. Data for the eligible pregnant population must be separately extractable.	Nonpregnant populations or mixed populations in which data for eligible pregnant migrant/refugee women cannot be separated.
Exposure	Testing or diagnosis for one or more prespecified sexually transmitted infections: Chlamydia trachomatis, Neisseria gonorrhoeae, syphilis (Treponema pallidum), Trichomonas vaginalis, genital herpes/HSV-2, human papillomavirus (HPV), hepatitis B virus (HBV) infection, HIV, HTLV-1, or Mycoplasma genitalium.	Studies that do not report an eligible infection separately or do not provide a valid numerator and denominator for the infection-specific prevalence estimate.
Comparator	No comparator is required for the primary prevalence question. When a nonmigrant comparator is reported, it will be extracted descriptively but is not required for inclusion.	None based solely on absence of a comparator.
Outcome	Primary: infection-specific prevalence, defined as the number of eligible pregnant migrant/refugee women with a specified infection divided by the number tested for that infection. Secondary: participant-level prevalence of “any STI” only when explicitly reported by the study using unique individuals.	Composite “any STI” prevalence derived by summing infection-specific cases, or outcomes without a defined eligible denominator.
Study design	Cross-sectional studies; cohort studies with prevalence data at a defined assessment; and population-based, registry, or surveillance reports with a defined source population, sampling/census approach, and denominator.	Case reports, case series, case-control studies, qualitative studies, editorials, commentaries, narrative or systematic reviews, and reports without a valid prevalence denominator.

Inclusion and exclusion criteria for selecting studies on the prevalence of sexually transmitted infections among pregnant migrant, refugee, asylum-seeking, undocumented, internally displaced, or otherwise displaced women. Eligibility criteria are organized by population, exposure or migration context, outcomes, study design, setting, language, publication type, and handling of duplicate data. Abbreviations: HBV, hepatitis B virus; HIV, human immunodeficiency virus; HPV, human papillomavirus; HTLV-1, human T-cell lymphotropic virus type 1; STI, sexually transmitted infection.

**Table 2:** Operational definitions.

Term	Definition
Migration [12]	Movement away from a usual place of residence, within or across borders, for a range of reasons and durations (temporary or permanent).
Migrant [12]	Umbrella term for a person who moves away from their usual residence (within a country or across an international border), regardless of legal status or motive.
International migrant [13]	Person who moves to a country other than their usual residence, commonly operationalized in statistics as being outside the usual country of residence for ≥1 year.
Internal migrant [13]	Person who migrates within a country, moving away from the usual residence to another region/area of the same country.
Immigrant [12]	From the destination-country perspective: a person who arrives in a country other than their country of origin to establish residence.
Emigrant [12]	From the origin-country perspective: a person who leaves their country of origin to reside elsewhere.
Refugee [14]	Person outside their country of nationality who is unable/unwilling to return due to a well-founded fear of persecution (classic Convention framing, often repeated verbatim in peer-reviewed literature).
Asylum seeker [12]	Person seeking international protection whose claim for refugee status has not yet been finally decided by the receiving state.
Internally displaced person (IDP) [13]	Person forced to flee their home (e.g., conflict/violence/human-rights violations) without crossing an international border.
Displaced person [13]	Broad, non-legal umbrella term for people forced to leave habitual residence; may include IDPs and refugees depending on context.
Forced migration / forced displacement [13]	Movement driven primarily by coercion/compulsion (e.g., conflict, persecution, violence, disasters), rather than voluntary choice.
Irregular migrant [13]	Person who enters, stays, or works in a country without required authorization under immigration laws.
Camp (refugee camp) [15]	A spatially bounded site of organized settlement/governance used to host displaced populations, sometimes becoming protracted.
Transit migrant / migrant in transit [12]	Person moving through an intermediate location en route to another intended destination; terminology is used inconsistently and is not a standardized legal category.

Operational definitions of key migration- and displacement-related terms used in this study. Definitions were adapted from the cited sources (references [12–15]. Abbreviations: IDP: internally displaced person.

### Search strategy

The literature search will be conducted in MEDLINE (via Ovid), Embase.com, Scopus, Web of Science, the Virtual Health Library, and the WHO Global Index Medicus. A preliminary strategy for Embase.com (S1 Table in S1 File) was developed collaboratively with a trained medical specialist librarian using controlled vocabulary and free-text terms for pregnancy, migration or displacement, and STIs. No restrictions will be applied by language, publication date, or geographic location. Database limits will exclude animal-only records and publication types that cannot provide eligible peer-reviewed primary prevalence data, including conference abstracts, letters, notes, editorials, preprints, dissertations, unpublished reports, institutional reports, national surveillance summaries, and ecological or aggregate population-level reports. No separate grey-literature search will be conducted. Studies in languages other than English or Spanish will be translated using appropriate translation support or native speakers when necessary. Search syntax will be adapted to each database and all strategies, dates, and limits will be documented.

### Screening

The study selection will be conducted in two phases and performed by two independent reviewers: In the first phase, titles and abstracts will be screened independently by both reviewers to identify potentially eligible studies. Any disagreement will be resolved through discussion, and if consensus cannot be reached, a third reviewer will be consulted. In the second phase, full-text articles of potentially eligible studies will be retrieved and assessed against the inclusion and exclusion criteria. Reasons for exclusion will be documented for all full-text articles that are excluded at this stage. The selection process will be documented using a PRISMA 2020 flow diagram, including the number of records identified, screened, excluded (with reasons), and included in the final review.

### Data extraction

Data will be independently extracted by two reviewers using a prespecified Microsoft Excel form (S2 Table in S1 File). Before formal extraction, both reviewers will pilot-test the form on a small sample of eligible studies to identify ambiguous, missing, or redundant fields. The form will then be refined by consensus, and any subsequent modifications will be documented. Formal extraction will be performed independently in duplicate. Disagreements will be resolved through discussion or consultation with a third reviewer. Extracted variables will include study and participant characteristics, migration category, source-population denominator, infection-specific numerators and denominators, diagnostic methods, biological specimens, prevalence period or assessment time, and information required to identify overlapping populations or duplicate reports.

### Risk of bias assessment

Risk of bias will be independently assessed by two reviewers using the Joanna Briggs Institute (JBI) Critical Appraisal Checklist for Studies Reporting Prevalence Data [16]. The checklist evaluates the appropriateness of the sample frame and sampling method, adequacy of sample size and coverage, description of participants and setting, validity and reliability of condition measurement, statistical analysis, and response rate.

Each JBI item will be rated as Yes, No, Unclear, or Not applicable. No aggregate numerical quality score will be calculated.

Disagreements will be resolved by consensus and, if necessary, consultation with a third reviewer. Item-level judgments and supporting rationale will be reported. For sensitivity analyses, studies with major risk-of-bias concerns in critical prevalence domains will be excluded to assess the robustness of pooled estimates. Risk-of-bias judgments will also inform interpretation of heterogeneity and certainty of evidence.

### Certainty of evidence

Certainty will be assessed separately for each infection-specific pooled prevalence estimate using a GRADE-informed approach adapted to prevalence evidence. This adaptation is informed by guidance for absolute event-rate or overall-prognosis estimates [17] and by methodological work on prevalence reviews [18]. Because no formal GRADE guidance is dedicated specifically to prevalence systematic reviews, we will explicitly report the adaptation and the rationale for every judgment. Observational prevalence evidence will begin at low certainty because observational sampling is the appropriate design for estimating prevalence. Two reviewers will independently consider five downgrading domains: risk of bias, inconsistency, indirectness, imprecision, and publication bias. Indirectness will address departures from the target population, migration context, diagnostic definition or test, and prevalence time frame. Imprecision will be judged from the width of the 95% confidence interval, the amount of information contributed, and whether the interval includes materially different prevalence estimates. Certainty will be rated as high, moderate, low, or very low, with disagreements resolved by consensus or a third reviewer.

### Data analysis and synthesis

#### Descriptive analysis.

Included studies will first be synthesized descriptively. We will summarize study design, setting, sampling strategy, eligibility criteria, source population, sample size, migration definition, maternal age, gestational age or trimester, country of origin and host country, and access to antenatal care when reported. For each infection, we will tabulate the diagnostic method, specimen, case definition or positivity threshold, number tested, number positive, prevalence estimate, confidence interval, and prevalence period or assessment time. We will also document missing or unclear data, potential population overlap, risk-of-bias judgments, and assumptions made during data handling. This descriptive synthesis will determine whether infection-specific quantitative pooling is clinically and methodologically appropriate.

#### Quantitative synthesis.

The primary unit of analysis will be an independent study population for a specific infection. When at least three comparable independent estimates are available for the same infection, we will conduct an infection-specific meta-analysis. A study that reports multiple STIs may contribute one estimate to each relevant infection-specific meta-analysis, but those correlated infection estimates will never be combined into a single pooled “overall STI” estimate. A composite prevalence of “any STI” will be analyzed separately only when the primary study explicitly reports the number of unique participants with at least one STI and the corresponding denominator. We will not derive this composite by summing infection-specific case counts because individuals may have coinfections.

For duplicate publications or overlapping cohorts, only one estimate per infection, underlying population, and prevalence period will be included in a given analysis, prioritizing the most complete report; nonoverlapping strata may be retained separately. When multiple eligible assessments are reported from the same cohort, the first or baseline antenatal assessment will be used for the primary prevalence analysis unless the report provides a clearly defined period prevalence across pregnancy. Infection-specific proportions will be synthesized with a random-effects model on the logit scale, with pooled estimates back-transformed to proportions. The Freeman–Tukey double-arcsine transformation will be examined as a sensitivity analysis rather than the primary method because its back-transformation can produce misleading results in some settings [19].

Forest plots will display study-specific and pooled prevalence estimates with 95% confidence intervals. Between-study heterogeneity will be described using Cochran’s Q, I^2^, and τ², and a 95% prediction interval will be reported when the number of studies permits a meaningful estimate. Heterogeneity will not be interpreted solely from fixed I^2^ cutoffs. Potential sources of heterogeneity will be explored using prespecified subgroup analyses and, when there are sufficient studies to avoid overfitting, meta-regression.

Prespecified subgroup analyses, when sufficient data are available, will examine:

- Geographic region or host-country region.
- Migration status (e.g., refugee, asylum seeker, undocumented migrant, internally displaced person).
- Diagnostic approach (laboratory-confirmed vs. syndromic or mixed ascertainment).
- Gestational trimester or timing of antenatal screening, when reported.

Additional subgroup analyses by study setting or report type will be considered only when prespecified categories are clinically meaningful and adequately represented.

Sensitivity analyses will assess robustness by excluding:

- Studies with major risk-of-bias concerns in critical JBI prevalence domains.
- Studies using syndromic diagnosis without laboratory confirmation, when the primary analysis includes such studies.

Small-study effects and possible publication bias will be explored for infection-specific meta-analyses with at least 10 studies using funnel plots on the analysis scale and an asymmetry test when statistically appropriate. Because funnel-plot asymmetry in meta-analyses of proportions can arise from mechanisms other than publication bias, results will be interpreted cautiously.

All analyses will be performed in R. The meta package will be used for meta-analysis of proportions, including the metaprop function and other validated R functions used when required for sensitivity analyses or meta-regression. The analysis code and analytic dataset will be made available with the completed review.

### Ethics and dissemination

This systematic review and meta-analysis will use aggregate data from previously published, peer-reviewed primary observational studies. No individual-level patient data will be collected and no participants will be recruited; therefore, institutional ethics approval is not required. The review will be conducted in accordance with the registered protocol. Any amendments will be documented transparently and reported in the final review. Findings will be disseminated through peer-reviewed publication and, where appropriate, academic or public-health forums focused on maternal health, migration, and sexually transmitted infections.

### Status and timeline of the study

This systematic review and meta-analysis is registered in PROSPERO under CRD420251085346. The current status is summarized in Table 3. The initial formal database searches have been completed and title-and-abstract screening is ongoing. Full-text screening, data extraction, risk-of-bias assessment, certainty assessment, quantitative synthesis, and manuscript preparation will follow the revised protocol. All amendments will be dated and reported in the final review and the PROSPERO record.

**Table 3:** Status and timeline of the systematic review and meta-analysis.

Review stage	Current status
Protocol development	Completed
PROSPERO registration	Registered; amendment to revised protocol planned before resubmission
Search strategy development	Completed
Formal database searches	Initial search completed; update search planned before final study selection
Title and abstract screening	Ongoing
Full-text screening	Planned
Data extraction	Planned
Risk-of-bias assessment	Planned
Certainty-of-evidence assessment	Planned
Data synthesis and meta-analysis	Planned
Manuscript preparation	Planned
Manuscript submission	Planned

Current status and projected timeline of the systematic review and meta-analysis stages.

## Discussion

Pregnant migrant and refugee women may face important barriers to antenatal care and STI screening because of displacement, legal precarity, socioeconomic vulnerability, and instability in healthcare access [20–23]. This systematic review and meta-analysis will address an important gap by producing infection-specific prevalence estimates rather than a single pooled STI measure, thereby preserving the epidemiologic meaning of distinct infections and avoiding double counting when one population is tested for multiple pathogens.

Important heterogeneity is anticipated across healthcare settings, migration categories, diagnostic methods, and the infections assessed. The revised analysis plan therefore prioritizes infection-specific synthesis, explicit handling of overlapping populations and coinfection, prevalence-appropriate study designs, and separate evaluation of diagnostic approach and migration context.

## Conclusion

This systematic review and meta-analysis will estimate infection-specific STI prevalence among pregnant migrant and refugee women and will characterize variation across migration contexts, regions, and diagnostic approaches. By separating infection-specific estimates and using explicit rules for overlapping populations and composite outcomes, the review aims to provide epidemiologically interpretable evidence for antenatal screening, migrant-health policy, and future research.

## Supporting information

S1 File Supporting Information captions are as follows: S1 Table. Preliminary search strategy developed for Embase.com, S2 Table. Prespecified data extraction instrument. S1 Checklist. PRISMA-P 2015 checklist.
(DOCX)
