# Supplementary material for: Prevalence of sexually transmitted infections among pregnant migrant and refugee women: Protocol for a systematic review and meta-analysis
Source: PLoS One. 2026 Oct 5;21(10):e0359424. doi: 10.1371/journal.pone.0359424 (PMC13637903; doi:10.1371/journal.pone.0359424)
Supplement: Supplementary file 1 [file pone.0359424.s001.docx]

Supplementary Material

Darío S López-Delgado,^1,3^ Carlos A Narváez,^2^ Gloria L Chapués-Andrade,^3^ Rosa Angela Ruiz Ibarra,^1^ Danilo Alejandro Solarte Ordoñez,^3^ Andrés Salas-Zambrano,^3^ Noah L Schroeder,^4^ Magda Gamba,^5,6^ Lysien I. Zambrano,^7^ Oscar H Franco,^8^ Alfonso J. Rodríguez-Morales,^9,10^

1. Hospital Universitario Departamental de Nariño, Pasto, Nariño, Colombia
2. Universidad Nacional Autónoma de Nicaragua, Managua, Nicaragua.
3. Universidad Cooperativa de Colombia, Campus Pasto, Colombia.
4. University of Florida, United States of America.
5. Central and University Library of Lucerne. Frohburgstrasse 3, 6005, Lucerne, Switzerland.
6. Faculty of Health Sciences and Medicine. University of Lucerne. Frohburgstrasse 3, 6005, Lucerne, Switzerland.
7. Department of Morphological Sciences, School of Medical Sciences, Universidad Nacional Autónoma de Honduras, Tegucigalpa 11101, Honduras.
8. Health and Bioethics, Julius Center for Health Sciences and Primary Care, Utrecht University, Utrecht, Netherlands
9. Faculty of Health Sciences, Universidad Científica del Sur, Lima 15074, Peru.
10. Grupo de Investigación Biomedicina, Faculty of Medicine, Fundación Universitaria Autónoma de las Américas-Institución Universitaria Visión de las Américas, Risaralda 660003, Colombia.

Table of Contents

[S1 Table. Preliminary search strategy developed for Embase.com	2](#_Toc237881458)

[S2 Table. Prespecified data extraction instrument	4](#_Toc237881459)

[S1 Checklist. PRISMA-P (Preferred Reporting Items for Systematic Review and Meta-Analysis Protocols) 2015 checklist	5](#_Toc237881460)

## S1 Table. Preliminary search strategy developed for Embase.com

| #1 | 'pregnant person'/exp OR 'pregnancy'/exp OR 'prenatal care'/exp OR 'expectant mother'/exp OR 'parturient'/exp |
| --- | --- |
| #2 | ((expect* NEXT/1 (women OR woman OR mother*)) OR ((labour OR labor) NEXT/1 presentation) OR pregnan* OR gestation* OR gravid* OR antenatal* OR 'ante natal*' OR prenatal* OR 'pre natal*' OR 'child bear*' OR 'childbear*' OR parturient*):ti,ab,kw |
| #3 | #1 OR #2 |
| #4 | 'migrant'/exp OR 'migration'/exp OR 'population migration'/exp OR 'forced migrant'/exp OR 'displacement (people)'/exp OR 'refugee camp'/exp OR 'asylum seeker center'/exp OR 'undocumented immigrant'/exp |
| #5 | ((conflict NEXT/1 (associated OR driven OR induced) NEAR/3 displacement*) OR ((forced OR 'war related' OR induced OR communit* OR people OR hous* OR population OR residential) NEAR/3 displacement*) OR (transit* NEAR/3 (condition* OR situation* OR camp OR camps))  OR migrant* OR emigrant* OR immigrant* OR migrat* OR immigrat* OR refugee* OR displaced OR 'asylum' OR 'residential mobility' OR transient* OR resettlement* OR 'undocumented status'):ti,ab,kw |
| #6 | #4 OR #5 |
| #7 | #3 AND #6 |
| #8 | 'infection'/mj OR 'sexually transmitted disease'/exp OR 'Treponema pallidum'/de OR 'Human immunodeficiency virus'/exp OR 'Human immunodeficiency virus infection'/exp OR 'Human immunodeficiency virus infected patient'/de OR 'Human T-lymphotropic virus 1'/exp OR 'hepatitis B'/exp OR 'hepatitis C'/exp OR 'Wart virus'/exp OR 'papillomavirus infection'/de OR 'ulcus molle'/exp OR 'Chlamydiaceae infection'/exp OR 'trichomoniasis'/exp OR 'Candida albicans'/exp OR 'vagina smear'/exp OR 'venereal disease reaction test'/exp OR 'HIV test'/exp OR 'enzyme linked immunosorbent assay'/de OR 'reagin test'/exp OR 'syphilis serology'/de OR 'seroprevalence'/exp OR 'Mycoplasma genitalium infection'/exp |
| #9 | ((('sexually transmi*' OR vener*) NEAR/3 (infect* OR disease* OR disorder*)) OR ('infectious disease*' NEAR/3 'vertical transmission') OR 'reproductive tract infection*' OR STI OR (human NEXT/1 ('immuno deficien*' OR immunodeficien* OR 'T lymphotropic' OR 'T Cell Lymphotropic' OR 'T Cell Leukemia') NEXT/1 virus*) OR (lymphadenopath* NEAR/3 (virus* OR retrovirus)) OR ((LAV OR 'lymphadenopathy associated') NEXT/1 antibodies) OR ('acquired immun*' NEAR/2 syndrome) OR 'HTLV III*' OR 'HTLV-III*' OR LAV-HTLV-III OR 'HTLV III LAV' OR HIV OR anti-HIV OR HIV-associated OR AIDS OR HIV-AIDS OR 'immunodeficien* associated virus' OR (infect* NEAR/4 (pallidum OR pallida)) OR syphili* OR 'great pox' OR 'lues' OR 'luetic disease' OR ((adult OR human) NEXT/1 ('t cell' OR t) NEXT/1 (leukaemia OR leukemia OR 'leukemia lymphoma' OR lymphotropic) NEXT/1 virus) OR 'HTLV 1' OR 'HTLV I' OR 'HTLV1'  OR (HBV NEAR/3 infect*) OR ((hippie OR injection OR serum OR 'type B' OR viral) NEXT/1 hepatitis) OR (('HB surface' OR 'anti australia' OR 'anti hepatitis') NEAR/2 (antigen* OR antibod*)) OR 'hepatitis B' OR 'Anti HBAg' OR ((HCV OR 'anti HCV') NEAR/3 (infect* OR antibod*)) OR 'hepatitis C' OR 'parenterally transmitted non a non b hepatitis' OR 'type C viral hepatitis' OR ((wart* OR condyloma OR papilloma) NEAR/3 (virus* OR infect* OR genital OR venereal)) OR (verruca NEAR/2 (virus* OR viral)) OR HPV OR 'HPV-16/18 infection' OR papillomavirus* OR (ducrey* NEAR/4 (disease* OR infect* OR streptobacillosis)) OR chancroid* OR chancre* OR 'ulcus molle' OR ((herpes OR HSV) NEAR/3 (genital* OR progenital* OR vener* OR vulvar OR 'type 2')) OR HSV-2 OR chlamyd* OR 'C. trachomatis' OR trichomon* OR 'T. vaginalis' OR gonorrh* OR gonococc* OR ((candida* OR C.) NEXT/3 (albican* OR stelladoidea OR oris)) OR ((albican* OR pinoyi OR psilosis) NEAR/3 (endomyces OR monilia OR oidium)) OR 'bacter* vaginosis' OR (genital* NEXT/1 (wart* OR ulcer* OR discharge*)) OR urethritis OR ((vagina* OR genital* OR test*) NEAR/3 (smear* OR swab*)) OR (('vener* disease*' OR regain OR treponema) NEXT/3 test*) OR VDRL OR (enzym* NEXT/2 immun* NEXT/2 assay*) OR 'enzyme linked immuno*' OR ELISA OR ((lues OR syphili*) NEXT/2 sero*)  OR (sero* NEXT/2 prevalen*) OR seroprevalence OR 'Mycoplasma genitalium' OR 'M. genitalium' OR (genitalium NEAR/2 mycoplasma*)):ti,ab,kw |
| #10 | #8 OR #9 |
| #11 | #7 AND #10 |
| #12 | #11 NOT ([animals]/lim NOT [humans]/lim) |
| #13 | #12 NOT ([Conference Abstract]/lim OR [Letter]/lim OR [Note]/lim OR [Editorial]/lim OR [Preprint]/lim) |

Preliminary reproducible search strategy developed for Embase.com using controlled vocabulary and free-text terms related to pregnancy, migration and displacement, and sexually transmitted infections. The strategy includes predefined limits for animal-only records and ineligible publication types and will be adapted as appropriate for the syntax and indexing of each additional database.

## S2 Table. Prespecified data extraction instrument

**Study characteristics**

Author(s)

Year of publication

Country/region and study setting

“Study design (peer-reviewed primary cross-sectional study or cohort study with a defined prevalence assessment)

Study period and dates of data collection

Unique study population/cohort/site identifier and information needed to detect overlapping reports

Population definition and eligibility criteria

Sampling or census method and source-population denominator

Sample size of eligible pregnant migrants/refugees

Funding or sponsorship source, if reported

**Participant characteristics**

Mean or median maternal age

Gestational age and/or trimester at STI testing, if reported

Country of origin and host country

Migration status (refugee, asylum seeker, undocumented migrant, internally displaced person, other displaced category)

Access to antenatal care, if reported

Educational level and housing/settlement status, if reported

**Infection-specific data**

STI(s) assessed, including HIV, syphilis, chlamydia, gonorrhea, trichomoniasis, genital herpes/HSV-2, HPV, HBV, HTLV-1, and Mycoplasma genitalium

For each STI: number positive (numerator)

For each STI: number tested in the eligible population (denominator)

For each STI: reported or calculated infection-specific prevalence and 95% confidence interval, when available

Prevalence definition and assessment period (point, defined period, first/baseline antenatal assessment, or other)

Diagnostic method/test algorithm and positivity threshold

Laboratory confirmation (yes/no/unclear) and biological specimen

Participant-level “any STI” composite: numerator, denominator, and study definition, only if explicitly reported using unique individuals

Coinfection information and combinations reported, if available

**Methodological and synthesis variables**

Duplicate/overlapping publication assessment and decision on which estimate is retained

JBI prevalence checklist item-level judgments (Yes/No/Unclear/Not applicable); no summed quality score

Missing or unclear data and contact with study authors, if undertaken

Stratified data by geographic region, migration category, gestational timing, diagnostic approach, or setting, when available

Nonmigrant comparator data, if reported (descriptive only; not required for inclusion)

Notes relevant to unit-of-analysis decisions, synthesis, or sensitivity analyses

Prespecified variables to be extracted independently by two reviewers from eligible studies. The instrument includes study and participant characteristics, migration status, infection-specific numerators and denominators, diagnostic methods, prevalence definitions, coinfection data, risk-of-bias information, potential overlapping populations, and variables required for subgroup and sensitivity analyses. The instrument will be pilot-tested before formal data extraction.

## S1 Checklist. PRISMA-P (Preferred Reporting Items for Systematic Review and Meta-Analysis Protocols) 2015 checklist

| Section and topic | Item No | Checklist item | Information Reported / Comment |
| --- | --- | --- | --- |
| ADMINISTRATIVE INFORMATION | | |  |
| Title: |  |  |  |
| Identification | 1a | Identify the report as a protocol of a systematic review | Yes – title identifies the report as a protocol |
| Update | 1b | If the protocol is for an update of a previous systematic review, identify as such | No – Not applicable |
| Registration | 2 | If registered, provide the name of the registry (such as PROSPERO) and registration number | Yes – PROSPERO CRD420251085346 |
| Authors: |  |  |  |
| Contact | 3a | Provide name, institutional affiliation, e-mail address of all protocol authors; provide physical mailing address of corresponding author | Yes – title page |
| Contributions | 3b | Describe contributions of protocol authors and identify the guarantor of the review | Yes – Authors’ contributions section |
| Amendments | 4 | If the protocol represents an amendment of a previously completed or published protocol, identify as such and list changes; otherwise, state plan for documenting important protocol amendments | Yes – Methods; protocol amendments will be dated, documented, and reported in PROSPERO and the final review |
| Support: |  |  |  |
| Sources | 5a | Indicate sources of financial or other support for the review | Yes – Funding section |
| Sponsor | 5b | Provide name for the review funder and/or sponsor | No – Not applicable |
| Role of sponsor or funder | 5c | Describe roles of funder(s), sponsor(s), and/or institution(s), if any, in developing the protocol | No – Not applicable |
| INTRODUCTION | | |  |
| Rationale | 6 | Describe the rationale for the review in the context of what is already known | Yes – Introduction |
| Objectives | 7 | Provide an explicit statement of the question(s) the review will address with reference to participants, interventions, comparators, and outcomes (PICO) | Yes – Objectives and Table 1 (PECOS) |
| METHODS | | |  |
| Eligibility criteria | 8 | Specify the study characteristics (such as PICO, study design, setting, time frame) and report characteristics (such as years considered, language, publication status) to be used as criteria for eligibility for the review | Yes – Methods, Section 3.1 and Table 1 |
| Information sources | 9 | Describe all intended information sources (such as electronic databases, contact with study authors, trial registers or other grey literature sources) with planned dates of coverage | Yes – Methods, Section 3.2; databases, citation searching. |
| Search strategy | 10 | Present draft of search strategy to be used for at least one electronic database, including planned limits, such that it could be repeated | Yes – Methods, Section 3.2 and S1 Table |
| Study records: |  |  |  |
| Data management | 11a | Describe the mechanism(s) that will be used to manage records and data throughout the review | Yes – Methods, Sections 3.2–3.4 |
| Selection process | 11b | State the process that will be used for selecting studies (such as two independent reviewers) through each phase of the review (that is, screening, eligibility and inclusion in meta-analysis) | Yes – Methods, Section 3.3 |
| Data collection process | 11c | Describe planned method of extracting data from reports (such as piloting forms, done independently, in duplicate), any processes for obtaining and confirming data from investigators | Yes – Methods, Section 3.4; the form will be pilot-tested and extraction performed independently in duplicate |
| Data items | 12 | List and define all variables for which data will be sought (such as PICO items, funding sources), any pre-planned data assumptions and simplifications | Yes – Methods, Section 3.4 and S2 Table |
| Outcomes and prioritization | 13 | List and define all outcomes for which data will be sought, including prioritization of main and additional outcomes, with rationale | Yes – Table 1 and Methods, Section 3.7.2; infection-specific prevalence is primary and any-STI composite is conditional on participant-level reporting |
| Risk of bias in individual studies | 14 | Describe anticipated methods for assessing risk of bias of individual studies, including whether this will be done at the outcome or study level, or both; state how this information will be used in data synthesis | Yes – Methods, Section 3.5 |
| Data synthesis | 15a | Describe criteria under which study data will be quantitatively synthesised | Yes – Methods, Section 3.7.2; pooling requires at least three comparable independent estimates for the same infection |
|  | 15b | If data are appropriate for quantitative synthesis, describe planned summary measures, methods of handling data and methods of combining data from studies, including any planned exploration of consistency (such as I^2^, Kendall’s τ) | Yes – Methods, Section 3.7.2; unit-of-analysis rules, random-effects logit synthesis, heterogeneity measures, and overlapping-population handling are prespecified |
|  | 15c | Describe any proposed additional analyses (such as sensitivity or subgroup analyses, meta-regression) | Yes – Methods, Section 3.7.2; subgroup, sensitivity, and meta-regression conditions are prespecified |
|  | 15d | If quantitative synthesis is not appropriate, describe the type of summary planned | Yes – Methods, Section 3.7.1; narrative and evidence-table synthesis will be used when pooling is inappropriate |
| Meta-bias(es) | 16 | Specify any planned assessment of meta-bias(es) (such as publication bias across studies, selective reporting within studies) | Yes – Methods, Section 3.7.2 |
| Confidence in cumulative evidence | 17 | Describe how the strength of the body of evidence will be assessed (such as GRADE) | Yes – Methods, Section 3.6; GRADE-informed prevalence adaptation specified |

Completed Preferred Reporting Items for Systematic Review and Meta-Analysis Protocols (PRISMA-P) 2015 checklist indicating where each applicable reporting item is addressed in the protocol.
